# Admission Heart Rate Variability Is Associated With Poststroke Depression in Patients With Acute Mild-Moderate Ischemic Stroke

**DOI:** 10.3389/fpsyt.2020.00696

**Published:** 2020-07-15

**Authors:** Lanying He, Jian Wang, Lijuan Zhang, Feng Wang, Weiwei Dong, Hao Yang

**Affiliations:** ^1^ Department of Neurology, The Second People’s Hospital of Chengdu, Chengdu, China; ^2^ Department of Neurology, The Second Affiliated Hospital of Chengdu College, Nuclear Industry, Chengdu, China; ^3^ Department of Neurology, First Affiliated Hospital, Chongqing Medical University, Chongqing, China; ^4^ College of Electrical Engineering, Institute of Electrical Technology, Chongqing University, Chongqing, China

**Keywords:** acute ischemic stroke, heart rate variability, fractal dimension, depression, Hamilton

## Abstract

**Background:**

Stroke has been shown to cause cardiac autonomic dysfunction. Depression is common complication after acute ischemic stroke (AIS). The purpose of this study was to investigate whether decreased heart rate variability (HRV) was related to poststroke depression (PSD) in patients with mild-moderate AIS.

**Methods:**

In this study, we assessed autonomic function of ischemic stroke patients within 72 h from symptom onset by fractal dimension (FD). 503 patients (mean age 65.93 ± 10.19) with mild-moderate AIS underwent FD test after admission. Depressive symptoms were assessed using 17-item Hamilton Depression Rating Scale (HDRS) at baseline (within 7 days) and 3 months. Depression were defined if HDRS >6 points. According to the data of FD, we investigated association with early-onset PSD status and 3-month PSD.

**Results:**

59.24% (293/503) of patients had early-onset PSD status at baseline, and 38.66% (184/476) of patients had PSD at 3 months. ROC curve analysis shown that the optimal cut point of FD for early-onset PSD status and 3-month PSD were FD ≤ 1.27 and FD ≤ 1.19, respectively. In fully adjusted models, higher NIHSS [adjusted odd ratios (OR), 1.15; 95% confidence interval (CI), 1.05–1.27; P=0.005], younger age (adjusted OR, 1.18; 95% CI, 1.01–1.13; P=0.046), and FD ≤ 1.27 (adjusted OR, 3.31; 95% CI, 2.23–4.92; P<0.001) were associated with increased risk of early-onset PSD status. Higher NIHSS (adjusted OR, 1.21; 95% CI, 1.09–1.43; P<0.001) and FD ≤ 1.19 (adjusted OR, 2.68; 95% CI, 1.79–4.03; P=0.000) were associated with increased risk of 3-month PSD.

**Conclusions:**

PSD is common after mild-moderate AIS and is highly correlated with decreased HRV, FD could serve as an objective tool to help in prediction of PSD.

## Introduction

Stroke is still the leading cause of disability in China (1). Stroke can lead to physical disability and emotional impairments. Poststroke depression (PSD) is the most common psychiatric implication of stroke. The prevalence of PSD in the first year after stroke was 41.8% (2), although the prevalence varied across studies due to methodological differences. PSD can have a negative influence on recovery, the mortality, quality of life of stroke survivors, and physical and cognitive functioning (3–6).

Heart rate variability (HRV), a measure of cardiac autonomic nervous system functioning, has emerged as a physiological indicator for emotional regulation and psychological well-being. It has been reported that stroke can cause cardiac autonomic dysfunction and HRV decreased, which reflecting poor parasympathetic regulation. In previous studies shown that decreased HRV was associated with PSD (7) and poor cognitive function (8). However, most of these studies used traditional linear HRV methods to predict PSD, and the sample size was small (9). Currently, prediction of PSD using non-linear HRV methods is still rare.

In physiological changes, the nonlinear methods may provide new insights into HRV (10–12). Healthy heart rates are slightly irregular and chaotic, fractal dimension (FD) can quantify the complexity of HRV, which is one of the most common nonlinear parameters (13). Compared with other classical linear methods, FD is more accurate and simpler. FD is related to dimensional complexity, and it estimates the self-similarity of a time interval in time series (13).

The objective of this study was to investigate whether decreased HRV by FD was associated with depression after mild-moderate AIS.

## Methods

The authors declare that all supporting data are available within the article.

### Study Population

The study included 503 consecutive mild-moderate AIS patients who were admitted to our stroke ward within 72 h of symptom onset (from December 2015 to December 2018). Stroke patients were diagnosed as AIS according to the WHO diagnostic criteria and was confirmed with the brain computed tomography (CT) or diffusion-weighted imaging (DWI) magnetic resonance imaging (MRI). The National Institutes of Health Stroke Scale (NIHSS) was used to measure the severity of Stroke. Eligible patients were admitted to our stroke unit. This study was approved by ethics committee. Written informed consent was obtained from all study participants.

### Inclusion Criteria

Patients were included in the study only if they fulfilled all the following criteria: (1). Aged from 18 to 90 years; (2). Admission for first-ever acute ischemic stroke within 24 hours; (3). Evidence of a single acute ischemic lesion consistent with clinical manifestations; (4). Finish speaking (No serious dysarthria); (5). Able to co-operate; (6). NIHSS ≤ 8; (7). All patients received standard therapy, which consisted of aspirin, lipid-lowering medications, and so on.

### Exclusion Criteria

The exclusion criteria were: (1) cardioembolic stroke; (2) prior neurological or psychiatric diseases; (3) cardiac (include acute myocardial infarction, a history of tachyarrhythmia/bradyarrhythmia or atrial fibrillation), long-term diabetes (>5 years after diagnosis) and those with evidence of neuropathy were also excluded; (4) severe pulmonary disease, renal failure (estimated glomerular filtration rate<30 ml/min 1.73 m^2^) and active malignancies were excluded; (5) Aphasia, hearing deficit, and drug abuse; (6) Cerebral hemorrhage, fever (≥38°C), hypoxia (arterial oxyhemoglobin saturation < 90%) on admission; (7) drug abuse; (8) did not appropriately communicate. All patients were followed up for 3 months.

### Data Collection and Scale Assessment

To rule out severe arrhythmia, including tachyarrhythmia/bradyarrhythmia or atrial fibrillation, all patients underwent routine 12-ECG (FX-7542, Fukuda, Japan) monitoring. Patients underwent routine 12-lead ECG and HRV monitoring between 9:00 am and 10:00 am on the next day after admission in a relaxed supine position, in a quiet room with an ambient temperature of 22°C, the recordings were undertaken under standardized conditions.

After the exclusion of severe arrhythmia, the patients underwent the portable 12-lead ECG (ECG-3312, Shanghai Yimu Medical instrument, China) examination, the RR intervals were recorded by the portable 12-lead ECG monitor with a sampling rate of 1000 Hz. The time scale was about 15 minutes, the beats were about 2000 beats. They were downloaded to the Precision Performance software (Chongqing Haikun Medical Instrument Co., Ltd). The software enabled the visualization of HR and the extraction of a cardiac period (RR interval) file in “txt” format. Following digital filtering complemented with manual filtering for the elimination of interference factors, including noise, premature beats, and artifacts. After this, all questionable portions were excluded, and only segments with >90% pure sinus beats were included in final analysis.

The chaos feature of RR intervals was represented by FD, which was used to quantify the complexity of the RR dynamic variation. Calculations were performed by means of an off-line software of the FD algorithm (Chongqing Haikun Medical Instrument Co., Ltd). FD was evaluated with the following equation, FD= logN (ϵ)/log (1/ϵ), where ϵ is scale and is used to measure RR interval, N (ϵ) is the number of measurement of RR interval (14–18). 512 continuous RR intervals were analyzed by inputting offline software, and FD value was calculated automatically for each subject (14, 18).

Patients screened positive for depressive symptoms, and whose diagnosis of clinical depression was verified by a diagnostic interview using DSM-5 criteria. PSD was assessed using the 17-item Chinese Hamilton Depression Rating Scale (HDRS) (19), each item on the questionnaire is scored on a 2- or 4-point scale depending on the item. Participants with a score of 0 to 6 points considered normal, 7–16 as mild depression, 17–24 as moderate depression, > 24 as severe depression. Early-onset PSD status was defined as HDRS>6 within 7 days after AIS. Persistent PSD was defined as PSD at 3 months. Experienced neurologists who implemented the scale assessment were blinded to the patients’ clinical information. They all received standardized training for the assessments, and interrater reliability reached an acceptable level.

### Statistical Analysis

Demographic characteristics and vascular risk factors were compared between no PSD and PSD groups in univariate analysis using Pearson χ 2 test, distributions of continuous variables were determined by the Kolmogorov–Smirnov test, while Mann–Whitney two sample test was applied in case of non-normal distributions. Receiver operating characteristic (ROC) curve analysis was used to determine the optimal cut point of FD for early-onset PSD status and 3-month PSD. We then performed logistic regressions analyses to determine the association between FD and early-onset PSD status and 3-month PSD, adjusting for all confounders (age, baseline NIHSS, sex, BMI, hypertension, current smoking, drinking, diabetes, hyperlipidemia, family history of stroke, etiological classification, infarct location, medications use). Results were expressed as adjusted odds ratios (aOR) with the corresponding 95% confidence interval (CI). The data was analyzed using SPSS software (SPASS 22.0). P values < 0.05 were considered as statistically significant.

## Results

### Characteristics of the Study Subjects

At baseline, 503 patients were identified, comprised 48.91% (246) men and 51.09% (257) women, and the mean age was 65.93 ± 10.19 years (41–84 years). In the study population, 323 patients had a history of hypertension, 150 had a history of diabetes, 250 had a history of hyperlipidemia, 138 patients smoke, the mean NIHSS on admission was 6.38 (± 2.02).

### Univariable Models for Predictors of Early-Onset PSD Status

In the early stage after stroke, 293 (293/503, 58.25%) had HDRS score >6 indicative of PSD status, 250 had HDRS score between 7 and 16 (mild PSD status), 38 had HDRS score between 17 and 24 corresponding to moderate PSD status, 5 had severe PSD status (HRDS score > 24). Significantly, those with early-onset PSD status had more severe stroke with mean NIHSS of 6.54 ± 1.98 compared with 6.16 ± 2.07 (P=0.030), younger age (65.13 ± 9.57 versus 67.05 ± 10.91, P=0.043),and lower FD value (1.17 ± 0.22 vs 1.43 ± 0.35, P<0.001). Other demographic characteristics were non-significantly different between those with early-onset PSD status (P>0.05) (**Table 1**).

**Table 1:** The demographic characteristics of early-onset PSD status.

	No PSD status group (210)	PSD status group (293)	OR (95% CI)	P*
Age, y (Mean SD)	67.05 ± 10.91	65.13 ± 9.57		**0.043**
NIHSS score, (Mean SD)	6.16 ± 2.07	6.54 ± 1.98		**0.030**
FD, (Mean SD)	1.46 ± 0.34	1.30 ± 0.27		**<0.001**
HDRS, (Mean SD)	3.90 ± 2.04	12.25 ± 4.23		**<0001**
FD ≤ 1.27, n (%)	73 (34.76)	181 (61.77)	4.13 (2.78–6.13)	**<0.001**
Females, n (%)	111 (52.86)	146 (49.83)	0.89 (0.62–1. 26)	0.503
Men, n (%)	99(47.14)	147 (50.17)	0.89 (0.62–1. 26)	0.503
BMI≥24 kg/m, n (%)	53 (25.24)	97 (33.11)	1.47 (0.99–2.18)	0.057
Hypertension, n (%)	132 (62.86)	191 (65.19)	1.11 (0.77–1.60)	0.591
Current smoking, n (%)	52 (24.76)	86 (29.35)	1.26 (0.85–1.89)	0.255
Drinking, n (%)	64 (30.48)	85 (29.01)	0.93 (0.63–1.37)	0.723
Diabetes, n (%)	55 (26.19)	95 (32.42)	1.35 (0.91–2.00)	0.132
Hyperlipidemia, n (%)	100 (47.62)	150 (51.19)	1.15 (0.81–1. 65)	0.429
Family history of stroke, n (%)	40 (19.05)	61 (20.82)	1.12 (0.72–1.74)	0.625
Etiological classification				
Large artery atherosclerosis, n (%)	91 (43.33)	134 (45.73)	1.10 (0.77–1.58)	0.593
Lacunar, n (%)	78 (37.14)	99 (33.79)	0.86 (0.60–1.25)	0.437
Other known causes, n (%)	1 (0.48)	6 (2.05)	4.37 (0.52–36.57)	0.138
Undetermined, n (%)	40 (19.05)	55 (18.77)	0.98 (0.63–1.54)	0.938
Infarct location				
Basal ganglia, n (%)	94 (44.76)	141 (48.12)	1.15 (0.80–1.63)	0.456
Brain stem, n (%)	34 (16.19)	40 (13.65)	0.82 (0.50–1.34)	0.428
Cerebellum, n (%)	15 (7.14)	18 (6.14)	0.85 (0.42–1.73)	0.655
Frontal lobe, n (%)	29 (13.81)	42 (14.33)	1.04 (0.63–1.74)	0.868
Parietal lobe, n (%)	16 (7.62)	15 (5.12)	0.65 (0.32–1.36)	0.250
Temporal lobe, n (%)	13 (6.19)	15 (5.12)	1.82 (0.38–1.76)	0.605
Occipital lobe, n (%)	9 (4.29)	22 (7.51)	1.81 (0.82–4.02)	0.138
Medications use				
Antiplatelet, n (%)	44 (20.95)	78 (26.62)	1.37 (0.90–2.09)	0.144
Antihypertensive, n (%)	114 (54.29)	158 (53.92)	0.99 (0.69–1.41)	0936
Lipid-lowering medications, n (%)	82 (39.05)	113 (38.57)	0.98(0.68–1.41)	0.913

*Comparison between no early-onset PSD status and PSD status groups. Continuous variables are expressed as mean ± standard deviation (SD). Baseline characteristics were compared between the 2 subgroups by univariate analysis using Pearson χ^2^, distributions of continuous variables were determined by the Kolmogorov–Smirnov test, Mann–Whitney two sample test was applied in case of non-normal distributions. Bold indicates P-values less than 0.05.

Receiver operating characteristic (ROC) curve analysis shows high accuracy for FD predict early-onset PSD status with AUC of 0.67 (95% CI 0.59 to 0.69), the optimal cut point of FD for early-onset PSD status was FD ≤ 1.27. Patients with FD>1.27 were 249 (49.50%), FD ≤ 1.27 in 254 (50.50%). Patients with FD ≤ 1.27 showed significantly higher prevalence of early-onset PSD status (181/254 vs 112/249; OR, 4.13; 95% CI, 2.78–6.13; P<0.001).

### Multivariable Models on the Association Between the Risk Factors and PSD Status

Unadjusted logistic regression analysis identified higher NIHSS score (OR, 1.10; 95% CI, 1.01–1.20; P=0.034), younger age (OR, 1.02; 95% CI, 1.00–1.04; P=0.038), and lower FD value (OR, 5.35; 95% CI, 2.94–9.74; P<0.001) as factors associated with a risk predictor of early-onset PSD status. In the multivariable logistic regression model, after adjusting for all confounders including (Model 1), higher NIHSS score[aOR, 1.15; 95% CI, 1.04–1.27; P=0.005)], younger age[aOR, 1.17; 95% CI, 1.00–1.13; P=0.047)], and lower FD value[aOR, 6.14; 95% CI, 3.23–11.67; P<0.001)] were associated with increased a risk of early-onset PSD status (**Table 2**). When FD ≤ 1.27 was entered into multivariate logistic regression (Model 2), FD ≤ 1.27 resulted to be associated with increased risk of early-onset PSD status (aOR, 3.31; 95% CI, 2.23–4.92; P<0.001), higher NIHSS (aOR, 1.15; 95% CI, 1.05–1.27; P=0.005) and younger age (aOR, 1.18; 95% CI, 1.01–1.13; P=0.046) were associated with increased risk of early-onset PSD status (**Table 2**). Using FD ≤ 1.27 to predict early-onset PSD status, the sensitivity was 61.77%, the specificity was 65.24%, positive predictive value was 71.26%; and negative predictive value was 55.02%.

**Table 2:** Multivariable models showing factors associated with early-onset PSD.

	OR (95% CI)	P*
Model 1		
Higher NIHSS	1.15 (1.04–1.27)	**0.005**
younger age	1.17 (1.00–1.13)	**0.047**
Lower FD value	6.14 (3.23–11.67)	**<0.001**
Model 2		
Higher NIHSS	1.15 (1.05–1.27)	**0.005**
younger age	1.18 (1.01–1.13)	**0.046**
FD ≤ 1.27	3.31 (2.23–4.92)	**<0.001**

*Multivariable adjusted for age, baseline NIHSS, sex, BMI, hypertension, current smoking, drinking, diabetes, hyperlipidemia, family history of stroke, etiological classification, infarct location, medications use. Bold indicates P-values less than 0.05.

### Univariable Models for Predictors of 3-Month PSD

During the study period, 15 patients had aphasia due to secondary stroke, 12 patients were lost to follow-up after discharge from the hospital, thus a total of 476 patients comprised the data as the available analysis set (no early-onset PSD status, N=195; early-onest PSD status, N=281), comprised 48.95% (233) men and 51.05% (243) women, and the mean age was 65.88 ± 10.14 years (39–80 years). In the study population, 313 patients had a history of hypertension, 144 had a history of diabetes, 235 had a history of hyperlipidemia, 127 patients current smoking, the mean NIHSS on admission was 6.41 (± 2.03). During the 3-month follow-up period, no patients died.

At 3 months, 58.01%(163/281) patients with early onest PSD status were diagnosed as 3-month PSD, 10.77% (21/195) patients with no early-onset PSD status were diagnosed with 3-month PSD, a total of 33.19% (184/476) of patients had PSD on 3 months, the mean ± SD score on the HDRS at baseline was 8.76 ± 5.42, decreasing to 6.85 ± 5.27 at 3 months, 184 (184/476, 38.66%) had HDRS score>6 indicative of depression, 153 had HDRS scores between 7 and 16 (mild depression), 29 had HDRS score between 17 and 24 corresponding to moderate PSD, 2 had severe depression (HRDS score > 24). Patients with 3-month PSD had higher NIHSS (6.73 ± 1.72 vs 6.11 ± 2.18, P=0.041), and lower FD value (1.29 ± 0.28 vs 1.40 ± 0.31, P < 0.001). Other demographic characteristics such as age, gender were non-significantly different between those with 3-month PSD (P>0.05) (**Table 3**).

**Table 3:** The demographic characteristics of PSD at 3 months.

	no PSD group (292)	3-month PSD group (184)	OR (95% CI)	P*
Age, y (Mean SD)	65.97 ± 10.18	65.72 ± 10.11		0.708
NIHSS score, (Mean SD)	6.11 ± 2.18	6.73 ± 1.72		**<0.001**
FD value, (Mean SD)	1.40 ± 0.31	1.29 ± 0.28		**<0.001**
HDRS, (Mean SD)	3.53 ± 2.67	12.12 ± 4.30		**<0.001**
FD ≤ 1.19, n (%)	91 (31.16)	98 (53.26)	2.52 (1.72–3.68)	**0.000**
Females, n (%)	150 (51.37)	93 (50.54)	0.97 (0.67–1.40)	0.861
Men, n (%)	142(48.63)	91 (49.46)	0.97 (0.67–1.40)	0.861
BMI≥24 kg/m, n (%)	86 (29.45)	54 (29.35)	1.00 (0.66–1.49)	0.981
Hypertension, n (%)	190 (65.07)	123 (66.85)	1.08 (0.73–1.60)	0.690
Current smoking, n (%)	79 (27.05)	48 (26.09)	0.95 (0.63–1.45)	0.816
Drinking, n (%)	79 (27.05)	59 (32.07)	1.27 (0.85–1.90)	0.241
Diabetes, n (%)	92 (35.51)	52 (28.26)	0.86 (0.57–1.28)	0.453
Hyperlipidemia, n (%)	137 (46.92)	98 (53.26)	1.29 (0.89–1. 87)	0.178
Family history of stroke, n (%)	55 (18.84)	36 (19.57)	1.05 (0.66–1.67)	0.844
Etiological classification				
Large artery atherosclerosis, n (%)	120 (41.10)	86 (46.74)	1.26 (0.87–1.82)	0.226
Lacunar, n (%)	113 (38.70)	61 (33.15)	0.79 (0.53–1.16)	0.221
Other known causes, n (%)	4 (1.37)	3 (1.63)	1.19 (0.26–5.39)	0.818
Undetermined, n (%)	58 (19.86)	32 (17.39)	0.85 (0.53–1.37)	0.502
Infarct location				
Basal ganglia, n (%)	135 (46.23)	87 (47.28)	1.04 (0.72–1.51)	0.823
Brain stem, n (%)	43 (14.73)	27 (14.67)	1.00 (0.59–1.68)	0.988
Cerebellum, n (%)	23 (7.88)	10 (5.43)	0.67 (0.31–1.45)	0.307
Frontal lobe, n (%)	42 (14.38)	24 (13.04)	0.89 (0.52–1.53)	0.680
Parietal lobe, n (%)	20 (6.85)	10 (5.43)	0.78 (0.36–1.71)	0.536
Temporal lobe, n (%)	13 (4.45)	13 (7.07)	1.63 (0.74–3.60)	0.222
Occipital lobe, n (%)	16 (5.48)	13 (7.07)	1.31 (0.62–2.80)	0.481
Medications use				
Antiplatelet, n (%)	72 (24.66)	44 (23.91)	0.96(0.62–1.48)	0.854
Antihypertensive, n (%)	168 (57.53)	94 (51.08)	0.77 (0.53–1.12)	0.169
Lipid-lowering medications, n (%)	103 (35.27)	77 (41.85)	1.32 (0.90–1.93)	0.150

*Comparison between no PSD and 3-month PSD groups. Continuous variables are expressed as mean ± standard deviation (SD). Baseline characteristics were compared between the 2 subgroups by univariate analysis using Pearson χ^2^, distributions of continuous variables were determined by the Kolmogorov–Smirnov test, Mann–Whitney two sample test was applied in case of non-normal distributions. Bold indicates P-values less than 0.05.

ROC analysis shows high accuracy for FD predict 3-month PSD with AUC of 0.72 (95% CI 0.56 to 0.69), the optimal cut point of FD for 3-month PSD was FD ≤ 1.19. Patients with FD>1.19 were 287 (60.29%), FD ≤ 1.19 in 189 (39.71%). Patients with FD ≤ 1.19 showed significantly higher prevalence of PSD (98/189 vs 86/287; OR, 2.52; 95% CI, 1.72–3.68; P<0.001).

### Multivariable Models on the Association Between the Risk Factors and 3-Month PSD

Unadjusted logistic regression analysis identified higher NIHSS score (OR, 1.10; 95% CI, 1.04–1.26; P=0.006) and lower FD value (OR, 3.46; 95% CI, 1.79–6.68; P<0.001) as factors associated with a risk predictor of 3-month PSD. In the multivariable logistic regression model, after adjusting for all confounders (Model 1), higher NIHSS (OR, 1.20; 95% CI, 1.08–1.33; P=0.001). and lower FD value (OR, 3.81; 95% CI, 1.89–7.67; P<0.001) were associated with increased risk of 3-month PSD (**Table 4**). When FD ≤ 1.19 was entered into multivariate logistic regression (Model 2), FD ≤ 1.19 (adjusted OR, 2.68; 95% CI, 1.79–4.03; P<0.001) and higher NIHSS (adjusted OR, 1.21; 95% CI, 1.09–1.34; P<0.001) were associated with increased risk of PSD (**Table 4**). Using FD ≤ 1.19 to predict 3-month PSD, the sensitivity was 53.26%, the specificity was 68.84%, positive predictive value was 51.85%; and negative predictive value was 70.03%.

**Table 4:** Multivariable models showing factors associated with 3-month PSD.

	OR (95% CI)	P*
Model 1		
Higher NIHSS	1.20 (1.08–1.33)	**0.001**
Lower FD value	3.81 (1.89–7.67)	**<0.001**
Model 2		
Higher NIHSS	1.21 ((1.09–1.34)	**<0.001**
FD ≤ 1.19	2.68 (1.79–4.03)	**<0.001**

*Multivariable adjusted for age, baseline NIHSS, sex, BMI, hypertension, current smoking, drinking, diabetes, hyperlipidemia, family history of stroke, etiological classification, infarct location, medications use. Bold indicates P-values less than 0.05.

## Discussion

In this study, we have investigated the association between FD value on admission and the development of depression at 7 days and 3-month PSD after acute mild-moderate ischemic stroke. The study showed that lower FD value was associated with the presence of PSD, and the risk of early-onset PSD status in patients significantly increased with FD ≤ 1.27, and the risk of 3-month PSD in patients increased with FD ≤ 1.19.

PSD is common among stoke survivors and can affect recovery. In this study, severity of depressive symptoms was assessed by the 17-item Chinese HDRS, the validity and reliability of the 17-item Chinese HRSD had been proven in previous studies (19), and interrater reliability was excellent, the item total score correlations were good, and the internal reliability was satisfactory. We found that a total of 58.85% of patients presented with PSD status at 7 days after mild-moderate AIS, during the 3 months follow-up, 58.01%(163/281) patients with early onest PSD status were diagnosed as 3-month PSD, 10.77% (21/195) patients with no early-onset PSD status were diagnosed with 3-month PSD, a total of 33.19% (184/476) of patients had PSD on 3 months, a total of 33.19% (184/476) of patients had PSD on 3 months, which was generally in line with previous publications (9). Therefore, our study showed that the patients with early-onset PSD status on 7 days after stroke could be temporary and reversible. But patients with early-onset PSD status were more likely to develop PSD at 3 months.

Cardiac dysautonomia is a common complication of stroke, the analysis of HRV is a well-recognized tool in the investigation of the autonomic control of the heart. Limited data, however, are available on the use of HRV in the assessment of the autonomic imbalance in patients after a prior stroke (4), and only traditional time linear methods have been considered (time and frequency domain methods), which measure the overall magnitude of RR intervals fluctuations around its mean value (20). However, it provides limited information about HRV, mostly because nonlinear mechanisms seem to be also involved in the genesis of heart rate dynamics (21). Among non-linear methods proposed so far to measure the fractal behavior of the HRV signal, FD has traditionally been approached following the chaos-theory, with the aim of modelling the attractor extracted from HRV sequences (13). In previous studies showed that it is feasible that the fractal dimension can be used for analyzing HRV (22–24).

In this study, FD was used to evaluate the status of autonomic function, investigate the relationship between FD and depression after AIS. Our results showed significant differences between groups of patients with PSD and no PSD. The depressive sample had lower FD values, younger age, higher NIHSS compared to no PSD group at 7 days, stroke severity and lower FD value remained persistently associated PSD at 3 months. This result can be interpreted as a reduced ability of the para-sympathetic nervous system to regulate the heart rate viavagal activity (25, 26). Patients with PSD may be limited in their ability to adapt their ANS activity to challenging environments and fail to evoke adaptive resources (27, 28). This may result in phenotypical depressive symptoms like exhaustion, fatigue, stress-reactivity, and disrupted sleep patterns (26). These findings present further evidence to previous findings of reduced HRV in depressive patients.

In this study, we validated the role of FD as a new tool for predicting early-onset PSD status and 3-month PSD. ROC curve analysis shown that the optimal cut point of FD for early-onset PSD status and 3-month PSD were FD ≤ 1.27 and FD ≤ 1.19, respectively. After adjusting for fully confounders, we found a significant association of FD ≤ 1.27 with increased risk of early-onset PSD status, and FD ≤ 1.19 with increased risk of 3-month PSD after AIS. To conclude, this study revealed that FD measured could be helpful in the prediction of PSD, especially when questionnaires could not be applied due to severe aphasia or cognitive disturbance.

In conclusion, our findings indicated that decreased FD in mild-moderate AIS was associated with increased risk of PSD. Lower HRV could be markers of higher vulnerability to develop PSD. FD may have potential predictive value in ischemic stroke.

## Limitation

However, these results must be interpreted cautiously and could not be generalized to all stroke patients due to several limitations. First, the patients were mild-moderate stroke, and severe stroke was excluded. Second, the absence of FD measurements at the 3-month, which did not clarify the causal relationship between cardiac autonomic function, depression after stroke. Third, although we adjusted for NIHSS score, which has been shown to correlate with infarction volume, we lacked data on infarction volume. Fourth, we lack data on the possible influence of the left and right insular stroke on FD and prognosis, respectively, because left and right insular lesion have different influence on the cardiac autonomic function. Fifth, we lack data on classical traditional HRV data in patients with PSD. Sixth, low sensitivity and specificity of FD are also one of the limitations of this study. In the future, the research will focus on how to improve the sensitivity and specificity of FD detection. In addition, larger multicenter longitudinal studies using neuroimaging techniques are needed to confirm our results and to better understand the pathophysiological mechanisms between poststroke autonomic function and mood disorders. However, despite these limitations, our research has the advantage that its large sample size, its analysis, including models adjusting for a wide variety of confounders, and a standardized way of measuring FD among patients.

## Data Availability Statement

All datasets generated for this study are included in the article/supplementary material.

## Ethics Statement

The studies involving human participants were reviewed and approved by Medical and Health Research Ethics Committee in Second People’s Hospital of Chengdu. The patients/participants provided their written informed consent to participate in this study. Written informed consent was obtained from the individual (s) for the publication of any potentially identifiable images or data included in this article.

## Author Contributions

LH was responsible for the concept and design of the study, data collection and analysis and the first draft of the paper and further manuscript. JW was responsible for concept and design of the study, the data analysis and interpretation. FW and LZ was responsible for the data analysis. WD was responsible for overseeing the concept and design of the study, the data analysis and interpretation, and HY was responsible for design software of FD in off-line compute. All authors contributed to the article and approved the submitted version.

## Conflict of Interest

The authors declare that the research was conducted in the absence of any commercial or financial relationships that could be construed as a potential conflict of interest.
